# Dengue hemorrhagic fever complicated with transient diabetic ketoacidosis: a case report

**DOI:** 10.1186/s13256-017-1476-z

**Published:** 2017-10-28

**Authors:** Chamara Dalugama, Indika Bandara Gawarammana

**Affiliations:** Department of Medicine, University of Peradeniya, Peradeniya, Sri Lanka

**Keywords:** Dengue hemorrhagic fever, Diabetic ketoacidosis, Pancreatitis

## Abstract

**Background:**

The increasing global prevalence of both dengue and diabetes may warrant closer observation for glycemic control and adapted fluid management to diminish the risk for a severe clinical presentation of dengue. Dengue illness is rarely known to precipitate diabetic ketoacidosis among diabetic patients. Both type 1 and type 2 diabetes increase the release of pro-inflammatory cytokines by various mechanisms and increase the risk of plasma leak in dengue fever. Acute pancreatitis is an atypical and rare presentation of dengue fever. We report a case of transient diabetic ketoacidosis in a previously well man which was challenging for the treating physician.

**Case presentation:**

A 26-year-old previously healthy Sri Lankan Sinhalese man presented to hospital with dengue hemorrhagic fever in compensated shock. He was found to have diabetic ketoacidosis and was managed with hydration and insulin infusion. Following recovery from dengue shock, his sugars normalized and ketogenesis stopped without exogenous insulin.

**Conclusions:**

Transient hyperglycemia with ketoacidosis, such as in our patient, has not been reported in the literature. Dengue virus inducing a transient pancreatitis during the viremic phase, however, is a possibility.

## Background

Sri Lanka, as a low-income country, is experiencing a growing chronic noncommunicable disease burden such as diabetes mellitus and a high incidence rate of dengue fever; understanding the comorbidity between the two disease groups is important in patient management. Dengue fever is rarely known to precipitate diabetic ketoacidosis among patients with diabetes [1–4]. Both type 1 and type 2 diabetes increase the release of pro-inflammatory cytokines by various mechanisms and increase the risk of plasma leak in dengue fever [5, 6]. Acute pancreatitis is an atypical and rare complication of dengue fever [7–9]. During hyperglycemia in dengue fever, urine output may not be a good guide of the volume status of the patient as the patient can be polyuric even during shock. Acidosis may be contributed both by the shock and ketones and may not reflect the severity of shock. We report a case of transient diabetic ketoacidosis in a previously well man which was diagnosed and managed during the critical phase of dengue fever and was challenging for the treating physician.

## Case presentation

A 26-year-old previously healthy Sri Lankan Sinhalese man presented to the Teaching Hospital, Peradeniya with fever, arthralgia, myalgia, and headache of 3 days’ duration. He complained of postural dizziness, nausea, and several episodes of vomiting since the previous day. At the same time, our patient has noticed increased frequency of passage of urine of 1 day duration. He denied any significant past medical history and no family history of diabetes mellitus among his first-degree relatives. He was a carpenter by profession and a nonsmoker and not an alcohol consumer.

On admission to the ward, our patient was conscious and rational, and he was complaining of thirst. He was severely dehydrated with cold, clammy peripheries and prolonged capillary refilling time. He was tachycardic with a pulse rate of 100 beats per minute. His blood pressure was 120/90 mmHg supine and 100/90 mmHg on standing. He had reduced breath sounds in his right lung base. An abdominal examination revealed a marked right hypochondrial tenderness with no clinically detectable free fluid.

On admission his packed cell volume was 55. An urgent complete blood count showed a platelet count of 15 × 10^3^, His hemoglobin level was 15 g/dL and white cell count 4.15 × 10^6^. His transaminases were elevated [aspartate transaminase (AST) > alanine transaminase (ALT)]. His serum creatinine level was 105 mmol/L and his serum electrolytes were normal. A bedside ultrasound scan of his abdomen showed free fluid in the hepatorenal pouch. Diagnosis of dengue hemorrhagic fever was made and fluid resuscitation started. Subsequently, the diagnosis was confirmed with a positive nonstructural protein 1 (NS1) antigen test result on admission and positive antibodies for both immunoglobulin M (IgM) and immunoglobulin G (IgG) on day 6 of his illness.

He was noticed to produce a considerable amount of urine contrary to the degree of dehydration. A random blood sugar test result was 296 mg/dL. At the same time, his urine ketone bodies were positive and arterial blood gas (ABG) showed compensated metabolic acidosis [bicarbonate 14 mmol/L, carbon dioxide partial pressure (PCO_2_) 23 mmHg and base excess of ˗-11] Diagnosis of diabetic ketoacidosis was made in the same patient. His serum amylase level was 95 U/L (normal range 22 to 80).

Our patient was initially started on intravenous normal saline bolus of 500 mL and fluids tailed off guided by clinical parameters and packed cell volume. He was started on soluble insulin infusion 0.1 u/kg (6 u per hour in this 60 kg man) and hourly blood sugar monitoring. Once his sugar level was less than 200 mg/dL, he was started on sugar-containing foods (glucose, biscuits, cake, and so on) and his sugar levels were maintained between 100 and 200 mg/dL. Insulin infusion rate was also tailed off according to the sugar level; we did not administer intravenous dextrose as it might have worsened the plasma leak from the dengue fever. Once his urine ketone bodies test results were negative, he was started on a regular dose of subcutaneous insulin and insulin infusion was stopped.

After recovery from dengue hemorrhagic fever, he was commenced on a routine diet and liberal fluids. After 2 days of recovering from the critical phase of dengue fever, his blood sugars were within normal limits without insulin, and his urine ketone bodies were persistently negative. His arterial blood gas showed normal bicarbonate. He was discharged on the fifth day after admission without insulin. He was reviewed in the ward 2 days after discharge and was asymptomatic. His fasting blood sugar was 98 mg/dL, postprandial blood sugar was 136 mg/dL, and anti-glutamic acid decarboxylase (GAD) antibodies were negative. Our patient was reviewed in the ward 5 months after his illness. He was back to his premorbid state. His glycosylated hemoglobin value was 5.8%, which was in the nondiabetic range, with a fasting blood sugar value of 92 mg/dL.

## Discussion

We report a case of a young Sri Lankan Sinhalese man presenting with dengue shock who was found to have a transient diabetic ketoacidosis and normalization of blood sugar with the resolution of viremia. A few cases of dengue illness precipitating diabetic ketoacidosis have been reported in the literature, but transient hyperglycemia with ketosis and acidosis during dengue viremia has not been reported in the literature.

Supradish *et al.* reported the case of a 16-year-old Thai girl who presented in dengue shock showing signs of severe dehydration and ascites [1], but who continued to have polyuria. She was found to have hyperglycemia, ketosis, and glycosuria. Diagnosis of diabetic ketoacidosis was made. Her volume replacement was adjusted according to the degree of dehydration, and signs of volume overload were monitored closely.

One review article concluded that those who reported diabetes were two and half times as likely to have dengue hemorrhagic fever [2]. The physiopathology of dengue hemorrhagic fever suggests amplification of the immune response due to the presence of heterotypic antibodies against a serotype of the dengue virus at the time of new infection [3, 4]. Type 1 diabetes mellitus is commonly associated with autoimmunity and the immune system may be persistently activated with signs of inflammation in tissues and capillaries, and is more likely to lead to inflammation and liberation of pro-inflammatory cytokines in tissues, particularly in the endothelium, which explains the higher risk of plasma leak in dengue fever [5]. Type 2 diabetes mellitus is a metabolic disorder that changes the anatomical and physiological integrity of the endothelium due to a permanent inflammatory condition caused by activation of T-lymphocytes, which leads to the release of pro-inflammatory cytokines such as gamma interferon (IFNγ) and tumor necrosis factor alpha (TNF-α), which increases the risk of dengue hemorrhagic fever [6].

Transient hyperglycemia with ketoacidosis, as in our patient, has not been reported in the literature. To date, there have been only a few case reports of acute pancreatitis complicating dengue fever from across the world [7–9]. Hyperlipasemia and enlarged pancreas have been known to occur in dengue fever; but acute pancreatitis is an atypical and rare presentation. In our patient his amylase level was marginally elevated but serum lipase measurement was not available. Dengue viruses inducing a transient pancreatitis or insulin resistance during the viremic phase are possible mechanisms.

## Conclusions

Dengue fever is hyperendemic in Sri Lanka. It can rarely present with various atypical endocrinologic manifestations. Both type 1 and type 2 diabetes increase the release of pro-inflammatory cytokines and increase the risk of plasma leak in dengue fever. Acute pancreatitis is a rare presentation of dengue fever. We report the first case of transient diabetic ketoacidosis during dengue hemorrhagic fever with normalization of sugars following resolution of the illness. Dengue virus inducing a transient pancreatitis or insulin resistance could be a possible mechanism.
